# Performance of GPT-4o in CT-based crista galli morphological classification: image-only versus morphometric dataset-driven approaches: a controlled comparative study

**DOI:** 10.1186/s12880-026-02618-3

**Published:** 2026-07-30

**Authors:** Erdal Komut, Serkan Günay, Ahmet Öztürk, Gurbet Yanarateş, Onur Karacif, Seval Komut

**Affiliations:** 1 Department of Radiology, Faculty of Medicine, Hitit University, Çorum, 19040 Turkey; 2 Department of Emergency Medicine, Faculty of Medicine, Hitit University, Çorum, Turkey; 3 Department of Radiology, Erol Olçok Education and Research Hospital, Çorum, Turkey

**Keywords:** ChatGPT, Computed tomography, Crista galli, Morphometric, Morphologic

## Abstract

**Background:**

The aim of this study was to examine whether ChatGPT’s performance in a morphological classification task based solely on a single CT slice and without access to any additional health record information varies depending on the inclusion of metric datasets.

**Methods:**

This study is based on a CT-based classification system in which categorized the crista galli morphometrically and morphologically. Paranasal CT (PNCT) images of 101 randomly selected patients who underwent PNCT were retrieved from the hospital’s database. The PNCT slices used by the radiologists for classification were uploaded individually to GPT-4o in a randomized patient order, and it was asked to perform morphological classification based solely on the CT images. The accuracy of the morphological classifications performed by GPT-4o based on the morphometric data and PNCT slices was statistically compared to the radiologists’ gold standard classifications.

**Results:**

GPT-4o correctly classified CG types in 27.72% of cases using PNCT slices, whereas its classification accuracy reached 100% when using the morphometric dataset. The accuracy of GPT-4o’s classification based on PNCT slices was significantly lower compared to its performance with morphometric data (*p* < 0.001). When examining the classification accuracy of GPT-4o based on PNCT slices by CG type, the highest accuracy was observed for the ossified type at 60%, while the lowest was for the tubular type at 6.06%.

**Conclusions:**

Although ChatGPT has demonstrated promising performance in diagnostic radiology when provided with textual or numerical datasets, in this controlled CT-based crista galli morphologic classification task, GPT-4o demonstrated substantially lower performance when relying solely on image input compared with structured morphometric data. These findings suggest that the performance of multimodal LLMs may strongly depend on structured information. Further studies involving broader diagnostic settings are required before conclusions regarding clinical applicability can be drawn.

## Background

Artificial intelligence (AI) has evolved considerably over the past decades and has found increasing applications in medicine [1]. Among the many existing AI models today, AI-powered chatbots also known as large language models (LLMs) have emerged as prominent tools that have proven their capabilities [2]. One of the most notable examples in this field is the Chat Generative Pre-trained Transformer (ChatGPT), a LLM developed by OpenAI (San Francisco, CA, USA), which gained significant popularity following its release in November 2022. Large language models, including ChatGPT, have attracted considerable interest for their potential applications in medicine [3, 4]. LLMs, in general, have the potential to enhance traditional approaches across a wide range of domains. However, the anticipated improvements in efficiency for clinical practice and the remarkable speed they promise in medical research have made ChatGPT particularly attractive in the field of medicine [5, 6]. The body of literature regarding ChatGPT is expanding rapidly, with studies focusing on its use in various areas such as medical diagnosis, treatment recommendations, patient education, and interpretation of medical reports [7–11]. 

The application of ChatGPT and other multimodal LLMs to medical image analysis and diagnostic support has attracted considerable interest in the field of radiology. As the literature on radiology continues to grow rapidly in areas including radiology education, patient education, routine screening programs, and report generation, newer ChatGPT versions with visual processing capabilities may pave the way for major transformations in clinical decision-making [12–15]. The interest in OpenAI’s popular product is fueled not only by its scientific or clinical implications but also by rational, paramedical factors such as the growing global demand for imaging, a shortage of radiologists, and the need for uninterrupted high-volume workflow [16–19]. While many studies have praised the benefits of multimodal LLMs and related applications, highlighting their potential to reduce diagnostic errors and improve clinical efficiency, some have raised concerns about AI’s potential to make unique, non-human-like errors [20, 21]. 

A major challenge in integrating AI directly into radiological workflows stems from its dependence on extensive datasets for comparison when analyzing images. Accurate radiologic diagnosis often requires evaluation of previous imaging of the same patient, review of medical records, and integration of information from both digital and human sources. Undoubtedly, accurate interpretation of radiological images also demands the analysis of variables such as tissue density, contrast variations due to patient hemodynamics, anatomical variants, and postoperative anatomical changes. Even if all relevant health history and variables are uploaded as a dataset, future literature will likely focus on how AI’s diagnostic accuracy compares to that of human radiologists. Nonetheless, current radiological research has largely concentrated on AI’s success in diagnosis and differential diagnosis from medical images, and its potentially augmentable and sustainable performance. Given that ChatGPT is fundamentally an LLM and chatbot, it remains unclear whether its success in radiology stems primarily from its image analysis capability or its proficiency in interpreting text and datasets.

Unlike previous studies, the aim of this research is to determine the extent to which ChatGPT requires a dataset in order to make accurate decisions within a Computed Tomography (CT)-based classification model. In other words, our study will examine whether ChatGPT’s performance in a morphological classification task based solely on a single CT slice and without access to any additional health record information varies depending on the inclusion of metric datasets. By doing so, we aim to contribute to future strategy and algorithm design for the integration of ChatGPT into clinical practice.

## Methods

### Ethical approval

The study was conducted in accordance with the Declaration of Helsinki and approved by the local ethics committee (Approval No: 2025/74, July 3, 2025). Informed consent was waived due to the retrospective anonymized study design.

### Crista galli classification by Komut et al

**Fig. 1:**
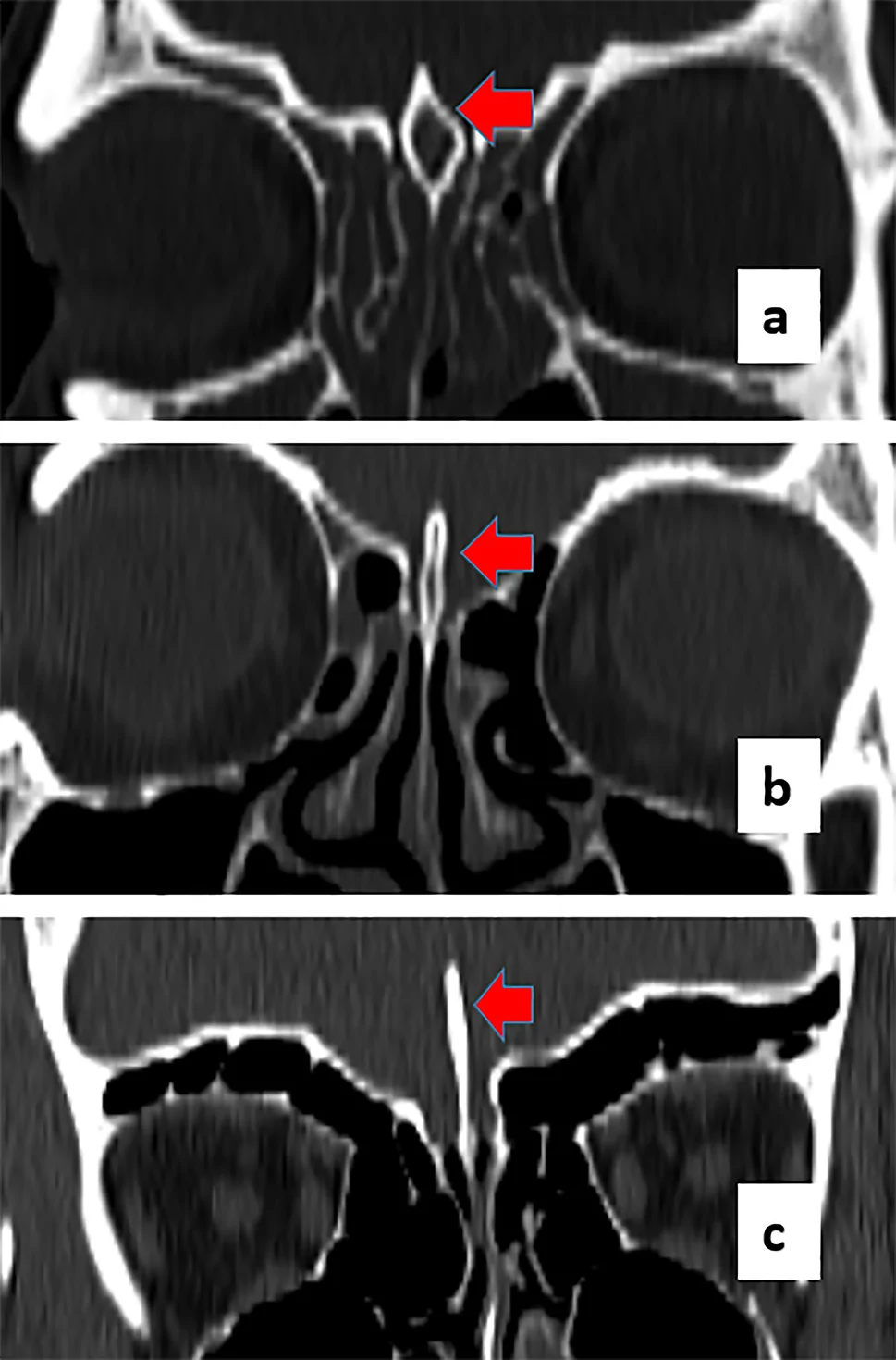
Examples of Komut’s morphological CG classification: **(a)** tear drop type (type 1), **(b)** tubular type (type 2), **(c)** ossified type (type 3). The red arrows indicate different types of CG on PNCT images

This study is based on a CT-based classification system in which Komut and colleagues categorized the crista galli (CG) morphometrically and morphologically [22]. In this classification, the dimensions of the CG and the presence of a cavitary component determine its type. If the width of the CG is greater than one-third of its height and contains a wide cavitary component, it is categorized as Type 1, or the “drop type,” on paranasal CT (PNCT) images (Fig. 1, a). If the width is less than one-third of the height and a cavitary component extends from the base to the apex, it is classified as Type 2, or the “tubular type” (Fig. 1, b). If the width is less than one-third of the height and there is no cavitary component, the CG is classified as Type 3, or the “ossified type” (Fig. 1, c).

This classification can be easily applied using a dataset created solely based on metric measurements and the presence or absence of a cavitary component. Moreover, as illustrated in Fig. 1, the classification can also be performed reliably using a single coronal PNCT slice focused on the CG. In this classification, morphometric measurements serve as proportional indicators rather than absolute numeric values. Thus, morphological classification shows a strong correlation with the metric dataset.

### Study design

**Fig. 2:**
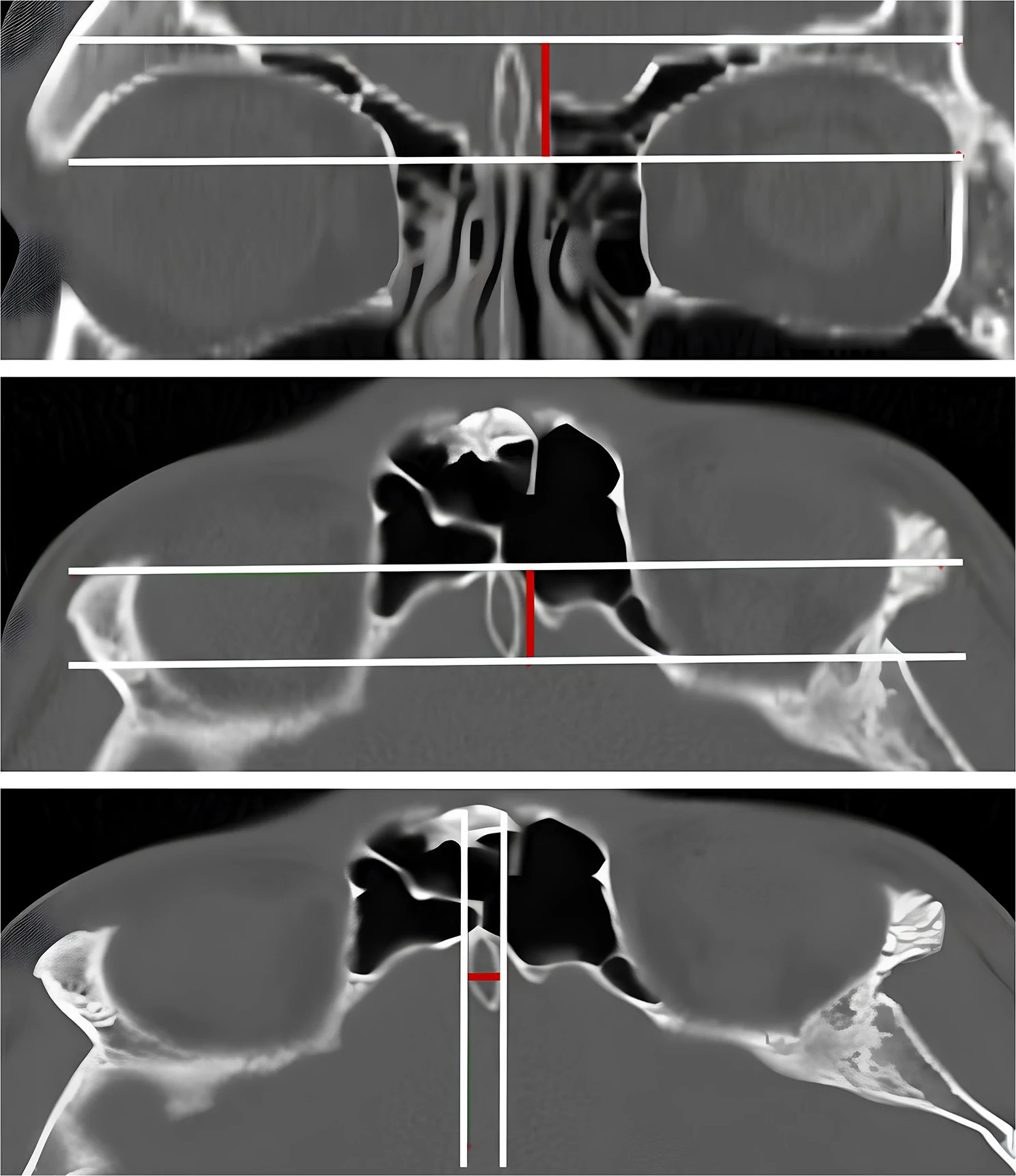
Demonstration of measurements for CG classification: (**a**) height (coronal plain); (**b**) length (axial plain); (**c**) width (axial plain). The red lines represent the measured distances

PNCT images of 101 randomly selected patients who underwent PNCT between July 1, 2024, and December 31, 2024, were retrieved from the hospital’s Picture Archiving and Communication System (PACS). Patients younger than 18 years of age were excluded. Cases with incomplete imaging data, severe motion or beam-hardening artifacts impairing crista galli evaluation, prior sinonasal or skull-base surgery, major craniofacial trauma, tumors affecting the anterior skull base or crista galli anatomy, or congenital craniofacial anomalies were also excluded. Following these quality-control procedures, eligible cases were randomly selected from the available examinations. Initially, all PNCT images were reviewed by a radiologist with 10 years of experience in CG measurements. After identifying the appropriate images, they were exported in DICOM format. Each image was assigned a unique identification number. The images contained no patient-related information, and no additional modifications were applied (e.g., annotations, arrows, averaging, cropping, or other image processing techniques). The same set of images was used for both the GPT-4o assessment and the radiologists’ measurements. Neither the radiologists nor GPT-4o had access to any clinical or case-related information associated with the images. Subsequently, two radiologists with six and eight years of neuroradiology experience measured the width, height, and length of the crista galli (CG) on coronal PNCT images and classified its type according to Komut’s classification (Fig. 2). The presence of any cavitary component was also documented alongside the morphometric measurements. Subsequently, a third radiologist with 12 years of neuroradiology experience independently performed the morphological classification based solely on PNCT slices and was blinded to the morphometric measurements and patient-related information. Complete agreement was observed among the three readers, with perfect interobserver agreement (Fleiss’ κ = 1.00, *p* < 0.001). Therefore, no disagreement-resolution process was required. These classifications were accepted as the reference standard for the study.

**Fig. 3:**
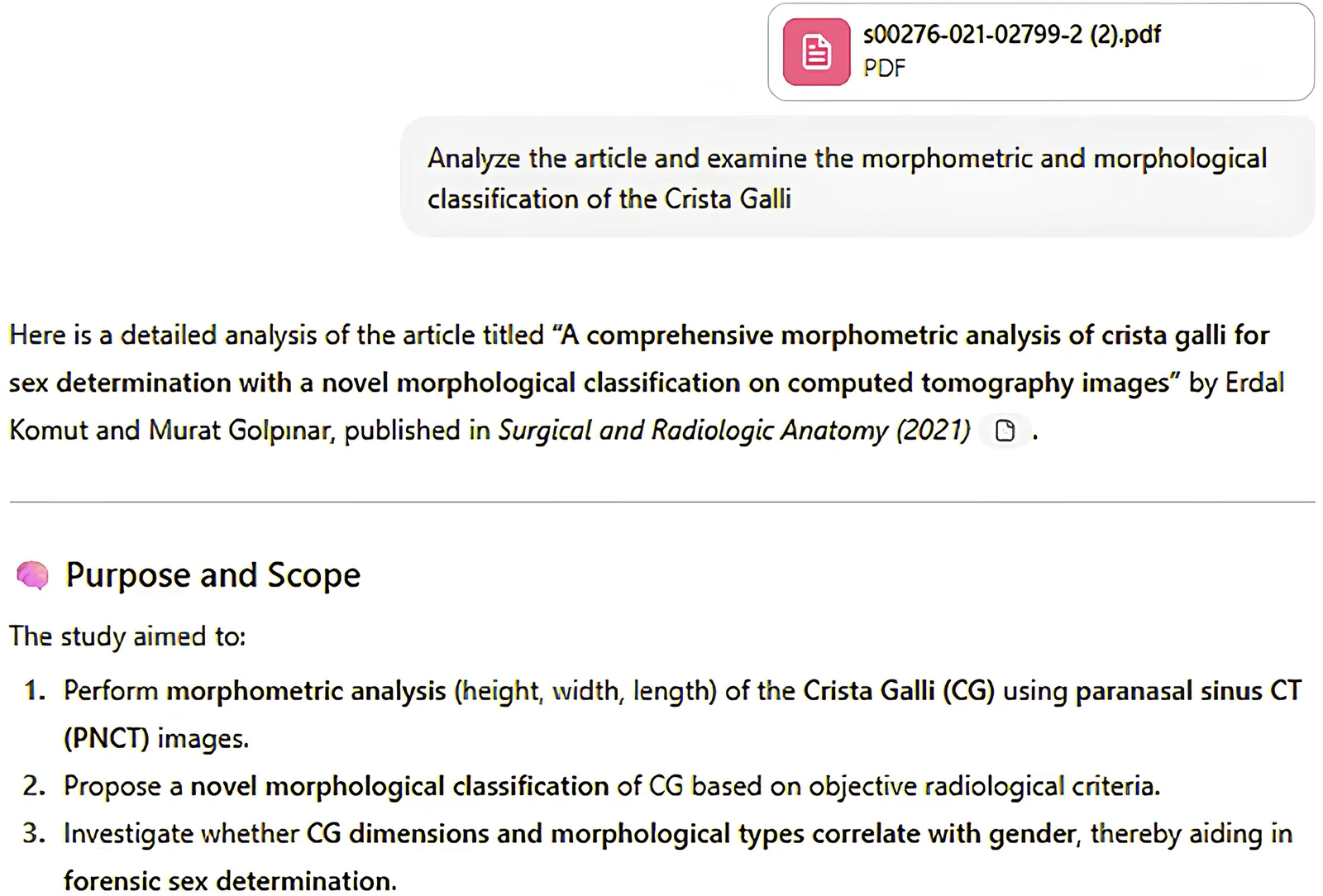
Screenshot showing the command given to ChatGPT-4o to analyze the article and learn Komut’s crista galli (CG) classification system

For evaluation purposes, the scientific article including the CG classification proposed by Komut et al. was uploaded into the GPT-4o model for analysis [22]. The command “Analyze the article and learn the morphometric and morphological classification of the CG” was given to GPT-4o. Upon completion of the analysis, the morphometric dataset was uploaded to GPT-4o, followed by the command “Classify the CG for each patient based on the dataset” (Fig. 3).

**Fig. 4:**
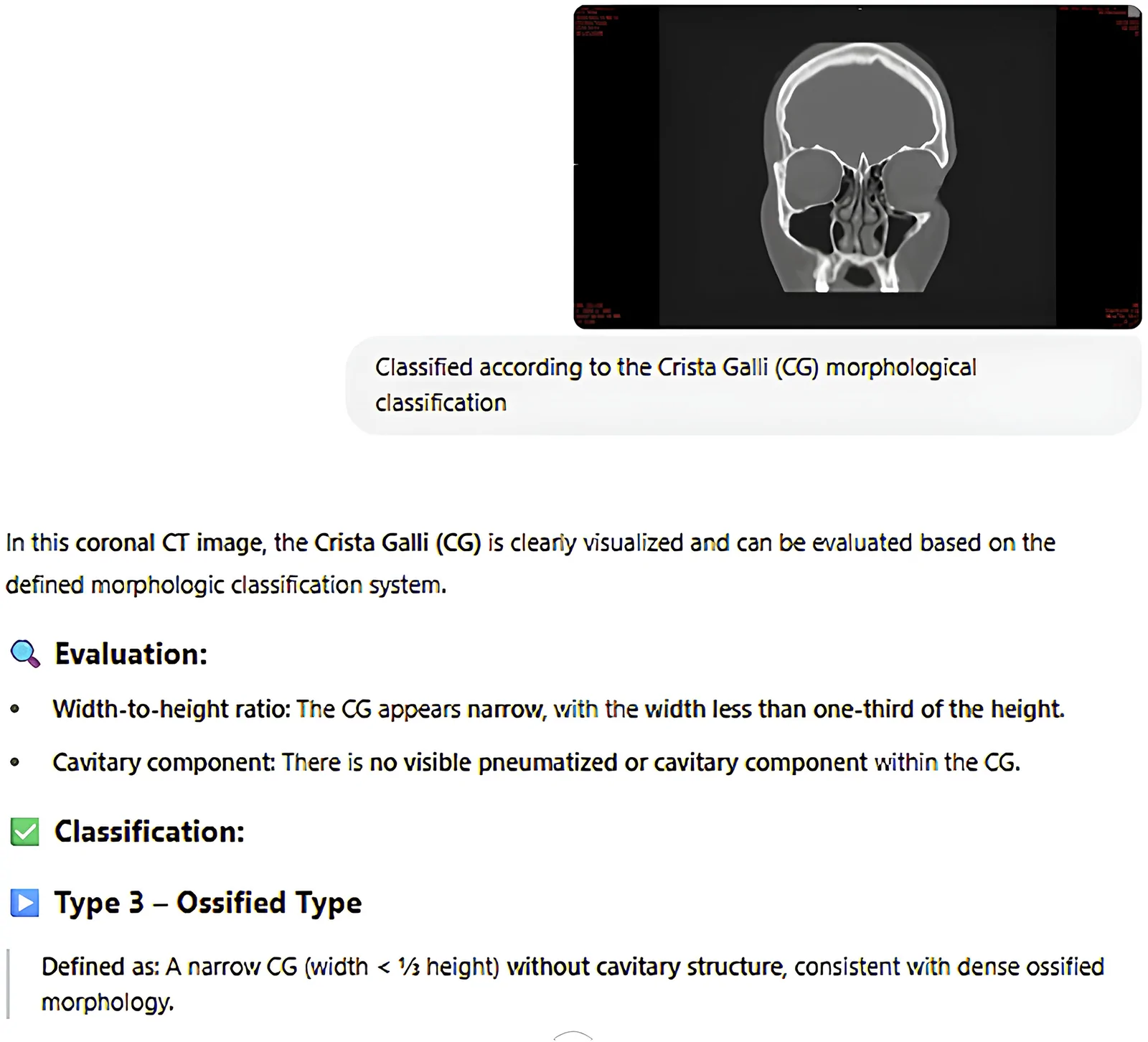
Representative failure case showing GPT-4o’s image-based morphological classification of the crista galli (CG). Although the reference standard classified the lesion as a tear-drop type (Type 1), GPT-4o incorrectly classified it as an ossified type (Type 3) based solely on the PNCT slice

**Fig. 5:**
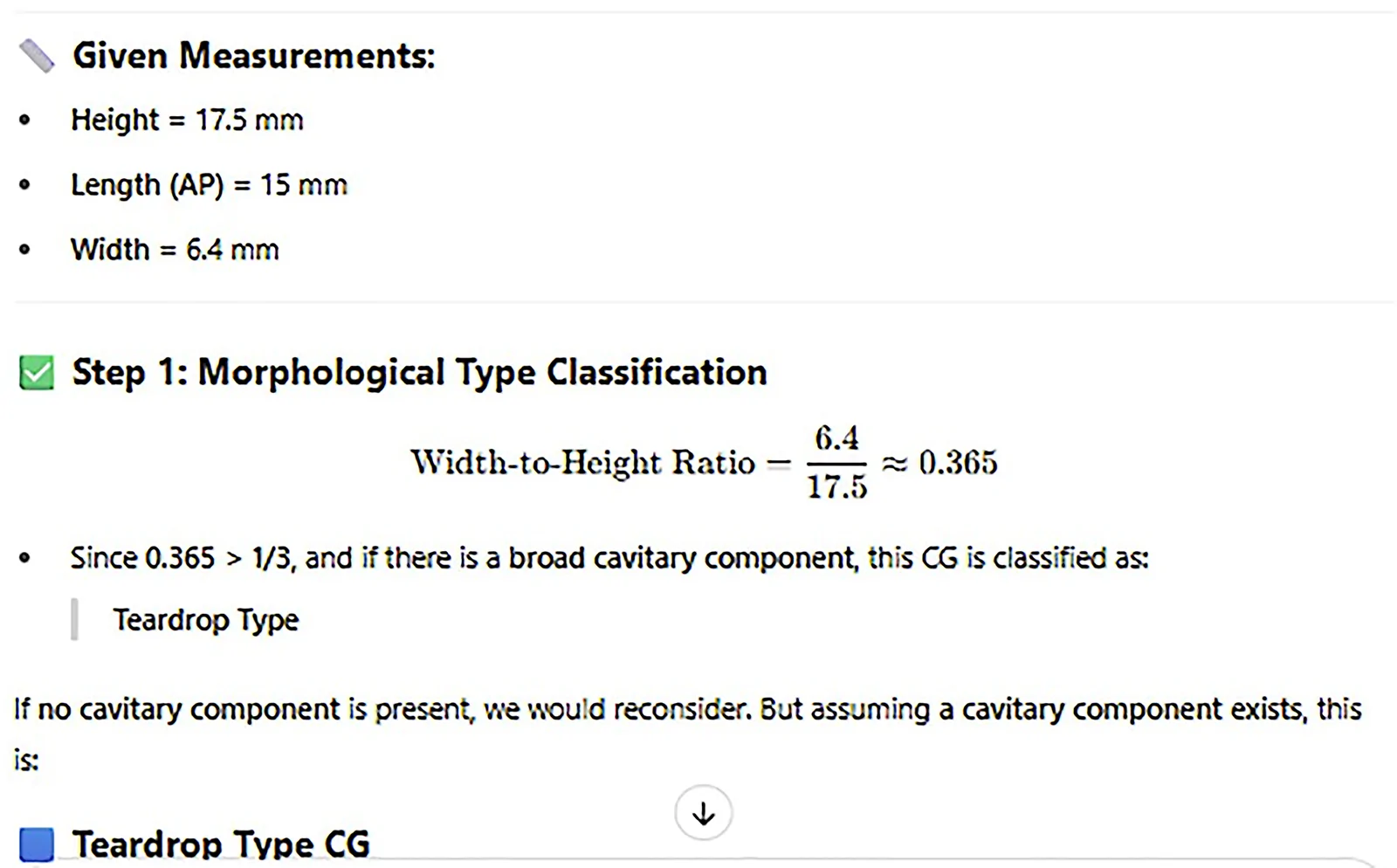
Representative successful case showing GPT-4o’s morphometric classification of the crista galli (CG) based on a structured textual dataset. In contrast to image-only analysis, GPT-4o correctly classified the crista galli according to the reference standard using morphometric information

The March 2025 version of GPT-4o (OpenAI, San Francisco, CA, USA), available through the ChatGPT interface, was accessed between August 10 and August 30, 2025, and used for all analyses. Identical prompts were used throughout the study. For each evaluation, a separate chat session was created, and morphologic and morphometric classifications were performed in independent chat sessions to minimize contextual carryover between cases and tasks. Each case was evaluated only once, and no responses were regenerated. The PNCT slices used by the radiologists for classification were uploaded individually in a randomized patient order. Images were uploaded in DICOM format. All images were anonymized and blinded to patient information and morphometric measurements. The DICOM images were displayed using the default bone window settings (window level, 500 HU; window width, 3000 HU) before upload to GPT-4o. No manual adjustment of these parameters was performed by the investigators. No additional image processing, annotations, or modifications were applied before uploading, and the images were presented in their original form. The model was instructed to classify the crista galli according to the previously provided classification system. Examples of GPT-4o’s patient-based analyses and responses, as per the given instructions, are shown in Figs. 4 and 5. The accuracy of the morphological classifications performed by GPT-4o based on the morphometric data and PNCT slices was statistically compared to the radiologists’ gold standard classifications.

### PNCT technique

The PNCT images were acquired using a 128-slice, Optima CT660 CT device (General Electric Medical Systems, Milwaukee, WI, USA). When taking the images, the patient was positioned supine and no sedation or contrast agent was used. Axial images were obtained according to following parameters: slice thickness, 1.25 mm; interval, 1.25 mm; kV, 100; mA, 120; pitch, 0.531; rotation time, 0.6 s; collimation, 20 mm; matrix, 512 × 512; FOV, 20 cm. Coronal images were obtained by reformatting the axial images with 1 mm slices thickness and 2 mm intervals, using a software program (Volume Viewer, General Electric Medical Systems, Milwaukee, WI, USA).

### Statistical analysis

Statistical analyses were conducted using IBM SPSS software, version 27. The normality of distribution was assessed using the Shapiro–Wilk test. Descriptive statistics for normally distributed variables were expressed as mean and standard deviation, while non-normally distributed variables were presented as median and interquartile range (IQR). McNemar’s test was used for pairwise comparisons of nominal variables. A p-value of < 0.05 was considered statistically significant. Interobserver agreement among the three radiologists was assessed using Fleiss’ kappa statistics.

The diagnostic performance metrics of GPT-4o in identifying crista galli morphologic types on CT images were calculated by comparing its classifications with the reference standard determined by expert radiologists. Sensitivity, specificity, positive predictive value (PPV), and negative predictive value (NPV) were computed using cross-tabulation analysis. Each morphologic type (tear-drop, tubular, and ossified) was analyzed separately.

## Results

A total of 101 patients were included in the study. Of these, 43.56% were female, and the median age was 33 years (IQR: 21). There was 100% agreement between the two radiologists—one performing morphometric measurements and the other evaluating only PNCT slices—regarding the classification. Based on Komut’s classification system, the radiologists identified 48 crista galli (CG) as tear-drop type (Type 1), 33 as tubular type (Type 2), and 20 as ossified type (Type 3) (Table 1).

**Table 1:** Descriptive analysis of study population

	All patients (*n* = 101)
Sex (female, %)	44 (%43.56)
Age (median, IQR)	33 (21)
Classification	
Tear-drop type (type 1) (n, %)	48 (%47.52)
Tubular type (type 2) (n, %)	33 (%32.67)
Ossified type (type 3) (n, %)	20 (%19.80)

GPT-4o correctly classified CG types in 27.72% of cases using PNCT slices, whereas its classification accuracy reached 100% when using the morphometric dataset. The accuracy of GPT-4o’s classification based on PNCT slices was significantly lower compared to its performance with morphometric data (*p* < 0.001, 95% CI = 0.6355–0.8101). Detailed results are presented in Table 2.

**Table 2:** Comparison of Responses Provided by GPT-4o Based on Visual and Quantitative Measurement Data

	Visual Data (n,%)	Quantitative Measurement Data (n,%)	Difference in 2 proportions	95% CI (lower-upper)	P value
Correct Responses Provided by GPT-4o	28 (27.72%)	101 (100%)	0.7228	0.6355–0.8101	< 0.001*

* According to McNemar test results

CI: confidence interval

When examining the classification accuracy of GPT-4o based on PNCT slices by CG type, the highest accuracy was observed for the ossified type at 60% (*n* = 12/20), while the lowest was for the tubular type at 6.06% (*n* = 2/33). The detailed distribution of GPT-4o’s classification accuracy across morphological types is provided in Table 3.

**Table 3:** Evaluation of correct responses provided by GPT-4o to visual data according to types

GPT-4o Classification (*n*, %)	Reference standard classification (*n*, %)			
	Tear-drop type (Type 1)	Tubular type (Type 2)	Ossified type (Type 3)	All types
Tear-drop type (Type 1)	**14 (29.17)**	5 (15.15)	8 (40.00)	27 (26.74)
Tubular type (Type 2)	6 (12.50)	**2 (6.06)**	0 (0.00)	8 (7.92)
Ossified type (Type 3)	28 (58.34)	26 (78.79)	**12 (60.00)**	66 (65.35)
All types	48 (100.00)	33 (100.00)	20 (100.00)	101 (100.00)

In our study, we also evaluated the diagnostic performance of GPT-4o in identifying morphologic types of the crista galli on CT images. For the tear-drop type, sensitivity was 29.7%, specificity 75.47%, PPV 51.85%, and NPV 54.05%. For the tubular type, sensitivity was 6.06%, specificity 91.18%, PPV 25%, and NPV 66.67%. For the ossified type, sensitivity was 60%, specificity 33.32%, PPV 18.18%, and NPV 77.14%. Detailed information is presented in Table 4.

**Table 4:** Evaluation of the diagnostic accuracy based on morphological types of crista galli

	Accuracy	Sensitivity	Specificity	+LR	-LR	PPV	NPV
Tear-drop type (Type 1)	53.47%	29.17%	75.47%	1.19	0.94	51.85%	54.05%
Tubular type (Type 2)	63.37%	6.06%	91.18%	0.69	1.03	25%	66.67%
Ossified type (Type 3)	38.61%	60%	33.32%	0.9	1.2	18.18%	77.14%

+LR= positive likelihood ratio, -LR= negative likelihood ratio, PPV= positive predictive value, NPV= negative predictive value

## Discussion

In our study, although ChatGPT-4o accurately classified all CG types when using morphometric data, its classification accuracy based solely on PNCT slices was limited to 27.7%. The substantially higher success rate of classification using the metric dataset, compared to the classification based on CT image analysis, highlights the current limitations of GPT-4o in this controlled CT-based morphologic classification task. However, in another study involving 100 neuroradiology cases where ChatGPT-4 was tasked with generating differential and final diagnoses, even after being provided with the complete clinical history of each case, the reported differential diagnosis accuracy was only 63%, and final diagnosis accuracy was just 50%. Interestingly, in that study, instead of actual radiologic images, imaging findings similar to clinical background data were uploaded to ChatGPT in the form of a dataset [23]. It is important to note that other studies have also reported variability in the diagnostic accuracy of ChatGPT depending on the anatomical region and the complexity of the underlying pathology [24, 25]. In contrast, in our study, ChatGPT-4o’s analysis based on the morphometric dataset was flawless. This result suggests that in the study by Horiuchi et al., published in Neuroradiology and based on “Case of the Week” materials from the American Journal of Neuroradiology, the diagnostic and preliminary diagnostic accuracy rates might have been lower if actual radiologic images had been uploaded to ChatGPT instead of imaging findings. In other words, the 63% preliminary and 50% final diagnosis accuracy reported by Horiuchi et al. may be attributable to the provision of imaging findings in a structured dataset format and/or the inclusion of the patients’ clinical history in the input to ChatGPT [23]. 

Supporting this idea, a study investigating ChatGPT’s ability to recognize urological pathologies based on CT and magnetic resonance imaging (MRI) slices obtained from Radiopaedia.com reported a final primary diagnosis accuracy of only 21% [26]. This rate is even lower than the 27.72% classification accuracy achieved by ChatGPT on CT images in our study. This discrepancy might be explained by the use of not only CT but also MRI images in the same study. Nonetheless, considering that the images in both of these studies were obtained from well-known online resources, a critical concern remains: whether similar diagnostic accuracies could be reproduced using original patient images in real-world clinical settings. This raises the broader issue of the reproducibility and generalizability of multimodal LLMs. Indeed, several studies have highlighted that the datasets on which AI-based algorithms are trained may still fall short in capturing and reflecting the variability seen in actual clinical environments [20, 27]. Therefore, we wish to re-emphasize that significant challenges still need to be addressed regarding the clinical integration of ChatGPT and other multimodal LLMs.

Several studies in the literature have reported that ChatGPT demonstrates variable success in identifying lesions or pathologies located in different anatomical regions [28]. In one study investigating ChatGPT’s diagnostic performance across various thoracic pathologies, the overall accuracy rate was reported as 56.76%, while the accuracy for identifying pathologies in the right and left hemithorax was 41% and 12%, respectively [28]. Moreover, in the same study, the accuracy rates for different lobes within the same hemithorax ranged widely, from 20% to 100% [28]. In another study evaluating 32 cases from the “Freiburg Neuropathology Case Conference,” different versions of GPT were tested with both clinical histories and radiological images provided. The results showed that GPT-4 achieved 22% accuracy for final diagnoses and 41% for differential diagnoses [29]. The authors of that study emphasized that textual input of radiologic image findings may be necessary to improve diagnostic performance when using ChatGPT as a diagnostic tool [29]. Another study, designed in the style of a radiology board examination using radiologic data and patient information, reported that ChatGPT achieved a diagnostic accuracy rate of 81% [30]. Undoubtedly, these findings collectively highlight the highly variable diagnostic performance of ChatGPT across different imaging modalities, anatomical systems, pathology types, and study designs. However, it may be generally inferred that diagnostic accuracy tends to improve in studies where datasets and textual clinical information are included as input, either in place of or in addition to radiologic images.

Most studies in current literature, however, are limited in that they do not allow a direct comparison of diagnostic performance between image-only analysis and analyses supplemented with textual or dataset inputs. Therefore, we believe that our study fills an important gap by evaluating a single radiologic outcome from both perspectives. Moreover, our study highlights that the primary challenge in this controlled classification task was not hallucination, but rather visual misclassification and the model’s dependence on structured information. Most classification errors involved cavitary subtypes, suggesting difficulties in recognizing subtle morphologic features and applying predefined classification rules. Furthermore, because repeated analyses were not performed, conclusions regarding response stability and reproducibility remain limited. These findings emphasize the importance of structured information and standardized evaluation protocols when assessing the performance of multimodal LLMs in radiologic classification tasks.

In our study, we also observed that GPT-4o demonstrated variable success rates when categorizing CG subtypes during morphometric classification. As shown in Fig. 1, ossified type (Type 3) CGs lack cavitary components or internal cavitations. With an accuracy rate of 60%, ChatGPT classified Type 3 CGs with the highest success, which may indicate that this subtype is the easiest to distinguish due to the absence of cavitary features. In contrast, the other two types contain cavitary components, and their main distinguishing features are the proportional width-to-height ratios observed in the coronal plane, along with the volumetric size of the cavitary component. GPT-4o was able to correctly classify 29.17% of tear-drop type (Type 1) CGs, whereas its performance in identifying tubular type (Type 2) CGs remained at only 6.06%.

In a recent study, the authors reported a sensitivity of 27.7% and a specificity of 60.47% for ChatGPT-4 V in the diagnosis of non-small cell lung cancer. In our analysis, the sensitivity and specificity rates observed for Type 1 CGs **(**29.7% and 75.47%, respectively) were relatively comparable to those values. However, the sensitivity markedly declined for Type 2 (6.06%) and increased for Type 3 (60%) CGs. The corresponding specificity rates in our study were 75.47%, 91.18%, and 33.32% for Types 1, 2, and 3, respectively. These findings collectively suggest that ChatGPT-4o struggles to accurately identify and quantify the cavitary component, which serves as a critical anatomic determinant in Komut’s CT-based classification system [28]. This limitation highlights the model’s relative inefficiency in recognizing subtle morphological features, particularly those related to the presence or volume of cavitary structures, despite its acceptable performance in morphometric differentiation.

Therefore, we interpret that GPT-4o, similar to its performance with morphometric data, is more successful in analyzing metric morphological differences than in detecting the presence of cavitary components. Indeed, while the only morphological difference between Type 2 and Type 3 is the presence or absence of a cavitary component, the model appeared to struggle more in distinguishing between these two than in differentiating either from the relatively short and wide Type 1.

However, when the presence of cavitary components was explicitly included in the dataset as written input, GPT-4o made no errors in classification. This supports the conclusion that morphometric data are analyzed far more effectively by GPT-4o than raw radiologic images. Supporting this view, a study examining MRI reports of 150 brain tumor patients prepared by neuroradiologists reported that GPT-4 achieved an 80% accuracy rate in producing final diagnoses [31]. That same study also highlighted that future studies focusing on textual datasets and radiologic images may offer more reliable insights into the clinical integration of LLMs [31]. To the best of our knowledge, no other studies in literature have reported results comparable to ours in this specific context. Therefore, we believe that our study will help illuminate the preclinical evaluation phase of ChatGPT and other large LLMs prior to their integration into medical practice, thereby contributing to the development of more robust diagnostic algorithms. In line with this, a study comparing GPT-4o’s diagnostic performance against real clinicians in identifying tibial plateau fractures from radiographs in 111 cases found an accuracy rate of only 27%. The primary limitation cited in that study was the lack of patient history and physical examination findings in the dataset provided to ChatGPT-4o [19]. Undoubtedly, the potential diagnostic value of textual or metric datasets in such studies should be quantitatively assessed in future comprehensive and multidimensional research.

In another study focusing on the multimodal diagnostic performance of GPT-4, a total of 221 cases were evaluated using X-ray, computed tomography (CT), and ultrasonography (US) images [32]. Although ChatGPT-4 successfully identified the radiologic modality in all cases, it showed varying degrees of error in localizing the anatomical region within each modality. Specifically, the model failed to identify the anatomical region in 39.1% of US cases and 3% of CT cases [32]. In a separate cohort study involving 100 cases of urological pathologies based on CT and MRI images, the authors reported a 21% accuracy in generating the primary diagnosis and 41% accuracy for the differential diagnosis. When ChatGPT was given additional prompts specifying the target organ or region of pathology after the primary diagnosis generation, the differential diagnostic accuracy increased from 30% to 62% for CT and from 52% to 62% for MRI. Similarly, the primary diagnostic accuracy improved from 21% to 35% [26]. 

These findings suggest that anatomical or organ-specific guidance, or the inclusion of textual/dataset-based cues, may significantly enhance ChatGPT’s performance in radiologic image analysis. It should be emphasized that GPT-4o is a general-purpose multimodal large language model rather than a dedicated radiology AI system specifically trained and validated for CT morphology classification. Therefore, the findings of the present study should not be interpreted as representative of medical imaging AI systems in general. In contrast, our study provided ChatGPT-4o with the original reference article describing the classification system in detail. Not only was the anatomical region specified (i.e., the CG), but the classification methodology itself was fully uploaded and explicitly instructed for the model to learn. In this context, the 27.72% classification accuracy achieved in our study despite these favorable conditions suggests that the performance would likely be even lower in the absence of such structured guidance, as would be the case with anonymous or publicly available classification tasks from online databases. This highlights a significant obstacle to consider in the broader implementation of AI for such tasks.

Interestingly, in the aforementioned study on urologic conditions, the authors noted that ChatGPT performed better with MRI than with CT images, yet they could not explain the underlying reasons [26]. This is particularly intriguing since MRI is generally considered more complex than CT in terms of sequence variations, contrast enhancement, and signal characteristics. In our study, ChatGPT-4o also struggled to apply a relatively simple rule the presence of a cavitary component as the sole distinguishing feature between Type 2 and Type 3 CGs. This suggests the existence of yet-unidentified variables influencing the learning and performance of AI-based algorithms. We believe that further investigation into these hidden factors is warranted and that the relevant literature must expand rapidly in the near future to better address these gaps in understanding.

With the increasing ability of ChatGPT-4o and other LLMs to process radiological images, the body of literature on this topic has grown at a remarkable pace. This rapid expansion has been driven not only by the potential advantages of AI-based medical algorithms such as increased diagnostic speed, reduced workload, and clinical decision support but also by the controversial threat posed by the generation of questionable academic content, particularly by anonymous LLMs like ChatGPT [33]. Radiology is considered one of the medical specialties most likely to be transformed by AI. However, the perspective that the diagnostic accuracy rates reported in the current literature may be subject to statistical pitfalls must be taken seriously before pursuing real-world clinical integration of multimodal LLMs [20]. The clinical integration of anonymous and highly advanced LLMs, such as ChatGPT, should not be rushed based on early yet potentially inflated expectations. Instead, multidisciplinary, prospective, and evidence-based research outcomes must be carefully scrutinized. Our findings demonstrate that ChatGPT, fundamentally an LLM, currently exhibits superior performance in text and dataset analysis compared with image-only interpretation in this controlled CT-based crista galli morphologic classification task. These results suggest that structured datasets may substantially enhance classification performance. However, conclusions regarding broader clinical applicability should be drawn cautiously, and future studies involving different anatomical regions, imaging modalities, and diagnostic settings are required to better characterize the role of multimodal LLMs in radiology.

A further limitation of this study is that each case was evaluated only once. Although repeated analyses across independent sessions could provide valuable information regarding the temporal reproducibility of GPT-4o outputs, the present study was specifically designed to evaluate the model’s diagnostic performance under routine real-world conditions at a predefined time point rather than its temporal stability. Furthermore, because GPT-4o is a continuously evolving proprietary model, analyses performed after completion of the original study cannot be assumed to represent identical experimental conditions to those of the original investigation. Therefore, repeated-session analyses extend beyond the predefined scope of the present study and should be investigated in future studies specifically designed to evaluate the temporal reproducibility of multimodal large language models.

In this study, original DICOM images were uploaded directly to GPT-4o without any manual preprocessing or adjustment of window width, window level, grayscale mapping, or other image-processing parameters by the investigators. However, because GPT-4o is a proprietary closed-source model, its internal image rendering, preprocessing pipeline, grayscale conversion, and display mechanisms cannot be independently verified. Consequently, the exact visual representation of the uploaded DICOM images received by the model remains unknown and should be regarded as an inherent black-box limitation of the present study. The objective of this study was not to investigate GPT-4o’s internal image-processing mechanisms but rather to evaluate the diagnostic performance of the model as experienced by end users under routine real-world conditions. In addition, no formal power analysis was performed to determine the sample size. Instead, patients were randomly selected from those who underwent imaging within the predetermined study period, without applying a statistically calculated sample size formula. Furthermore, the ChatGPT web interface does not provide user access to temperature or other randomness-related settings. Therefore, these parameters could neither be standardized nor reported, which may have affected the reproducibility of the model outputs. ChatGPT has demonstrated promising performance in diagnostic radiology when provided with textual or numerical datasets, In this controlled CT-based crista galli morphologic classification task, GPT-4o demonstrated substantially lower performance when relying solely on image input compared with structured morphometric data. These findings suggest that the performance of multimodal LLMs may strongly depend on structured information. Further studies involving broader diagnostic settings are required before conclusions regarding clinical applicability can be drawn. Our findings suggest that supplementing radiologic image analysis with structured datasets may enhance diagnostic performance in certain controlled settings. Nevertheless, the literature in this field must continue to evolve cautiously, through comprehensive, multidisciplinary investigations, in order to shed light on this promising yet complex frontier and help address the growing number of unanswered questions.

## Data Availability

The datasets generated and/or analysed during the current study are not publicly available due to patient confidentiality and institutional data protection policies but are available from the corresponding author on reasonable request.

## Abbreviations

AI: Artificial intelligence
CG: Crista galli
CT: Computed tomography
FOV: Field of view
GPT-4o: Chat Generative Pre-Trained Transformer, version 4-omni
IQR: Interquartile range
LLM: Large language model
MRI: Magnetic resonance imaging
NPV: Negative predictive value
PACS: Picture Archiving and Communication System
PNCT: Paranasal computed tomography
PPV: Positive predictive value
SPSS: Statistical Package for the Social Sciences
